# Waning immunity drives respiratory virus evolution and reinfection

**DOI:** 10.1093/emph/eoaf002

**Published:** 2025-01-31

**Authors:** James J Bull, Katia Koelle, Rustom Antia

**Affiliations:** Department of Biological Sciences, University of Idaho, Moscow, ID, USA; Department of Biology, Emory University, Atlanta, GA, USA; Emory Center of Excellence for Influenza Research and Response (CEIRR), Atlanta, GA, USA; Department of Biology, Emory University, Atlanta, GA, USA; Emory Center of Excellence for Influenza Research and Response (CEIRR), Atlanta, GA, USA

**Keywords:** antigenic evolution, influenza virus, mathematical model, SIR model, viral escape

## Abstract

Viruses differ in the number and types of host tissues in which they replicate. For example, systemically replicating viruses such as measles infect cells and tissues throughout the body, whereas respiratory viruses such as influenza viruses and coronaviruses replicate only in the respiratory tract. Reinfections with respiratory viruses are thought to be driven by ongoing antigenic immune escape in the viral population. However, this does not explain why antigenic variation is frequently observed in respiratory viruses and not systemically replicating viruses. Here, we argue that the rapid rate of waning immunity in the respiratory tract is a key driver of antigenic evolution in respiratory viruses. Waning immunity results in hosts with immunity levels that protect against homologous reinfection but are insufficient to protect against infection with an antigenically different (heterologous) strain. Thus, when partially immune hosts are present at a high enough density, an immune escape variant can invade the viral population even though that variant cannot infect solidly immune hosts. Invasion can occur even when the variant’s immune escape mutation incurs a fitness cost, although any such cost is likely to be short-lived from compensatory evolution. Thus, the mutant lineage may replace the wild type and, as immunity to it builds, the process will repeat. Our model provides a new explanation for the pattern of successive emergence and replacement of antigenic variants that has been observed in many respiratory viruses. We discuss our model relative to others for understanding the drivers of antigenic evolution in respiratory viruses.

## INTRODUCTION

Many respiratory viruses including influenza viruses, coronaviruses, and respiratory syncytial virus (RSV) present challenges for control because individuals are repeatedly infected throughout their lifetimes. Longitudinal cohort studies indicate that individuals can become reinfected with the same influenza virus subtype in months to years following a previous infection [14, 39]. Virological and serological studies on the four seasonal human coronaviruses also indicate that individuals are reinfected every few years [10, 15], and a recent review indicates that similar patterns of reinfection are observed in SARS-CoV-2 [32]. RSV reinfection has also been shown to commonly occur [16]. Frequent reinfection is not characteristic of all human viruses, however. For example, we are rarely re-infected with measles, yellow-fever, smallpox, mumps, and rubella, all of which are viruses that undergo systemic replication.

**Fig 1:**
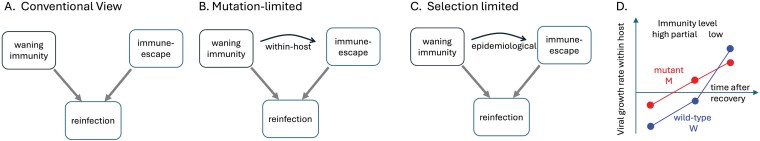
The relationship between waning immunity, immune escape, and reinfection in respiratory viruses. (a) A schematic depicting the conventional view of factors affecting host reinfection. Waning immunity refers to an individual’s decay in T-cell and antibody levels over time. Immune escape refers to genetic changes in the viral population that result in antigenic novelty. Here, an individual can become reinfected because their immunity has waned or because they are challenged with a strain that is antigenically different from their previous infection. Under this view, waning immunity and immune escape are independent factors that enable reinfection. (b) A schematic depicting an existing view [19] of the factors affecting host reinfection. Here, immune escape is not independent of waning immunity; rather waning immunity is a driver of immune escape in the viral population. This view specifically proposes that waning immunity generates partially immune individuals who, once infected, are more likely to generate immune escape variants. This model assumes a mutation-limited view of antigenic evolution. (c) A schematic depicting our view of factors affecting host reinfection. Here, as in (b), waning immunity is a driver of immune escape in the viral population. However, we propose that partially immune individuals act as hosts that enable the spread of immune escape variants by providing these variants with a selective advantage at the level of the host population. This model assumes a selection-limited view of antigenic evolution. (d) The effect of host immunity on within-host viral growth rates that is assumed by our model. Following infection with a wild-type virus, individuals transition over time from having high to partial to low immunity levels. Neither virus can grow in (or transmit from) hosts with high levels of immunity to the wild-type virus. Both viruses can grow in (and transmit from) hosts with low levels of immunity to the wild-type virus, but the mutant has a lower within-host growth rate due to the fitness cost of the antigenic mutation. This within-host growth disadvantage translates into a between-host transmission disadvantage owing to lower viral titers and/or more rapid clearance from the host. Only the mutant virus can grow in (and transmit from) partially immune hosts, due to the antigenic difference between the mutant virus and the wild-type virus. The growth rate of the mutant virus is lower in partially immune hosts than in hosts with low levels of immunity. As a result, the transmission rate of partially immune individuals infected with the mutant virus may be lower than that of individuals infected with the mutant virus who have only low levels of immunity.

The lack of reinfection observed for viruses that undergo systemic replication is generally thought to be jointly due to the persistence of high levels of immunity and viral antigenic stability. Why, then, can viruses that replicate only in the respiratory tract reinfect? It seems that the answer must lie either with waning host immunity, viral immune escape, or both (Fig. 1a). The effect of waning immunity on reinfection, classically modeled using an SIRS (susceptible-infected-recovered-resusceptible) framework, has been applied to respiratory viruses including influenza viruses [9, 44], and there is evidence that homologous immunity decays over time for this class of viruses [6, 14, 17, 25, 38]. Antigenic changes in viral populations are another obvious culprit underlying reinfection potential in respiratory viruses because these viruses commonly exhibit ongoing antigenic evolution [11, 22–24, 35, 40, 43]. While antigenic changes are both associated with and contribute to reinfection, it is not clear why respiratory viruses typically exhibit antigenic evolution while viruses undergoing systemic replication do not.

One possible explanation relies on variation in mutational tolerance across viruses: viruses that exhibit a greater degree of mutational tolerance may have more opportunity to evolve immune escape. In support of this explanation, the hemagglutinin (HA) surface protein of influenza viruses shows greater mutational tolerance than the HA surface protein of the measles virus [21, 42]. A second explanation invokes variation in the breadth of the immune response across viruses: viruses that elicit a narrow response to an immunodominant epitope may more easily evolve immune escape than viruses that elicit a broad response against multiple epitopes [18]. This explanation is supported by neutralization studies in measles virus showing that antigenic sites are serologically co-dominant [30], while for influenza viruses, the immune response appears to be directed at only one to two immunodominant epitopes [1, 46]. While both of these explanations are plausible and each have some empirical support, it is not clear why factors such as mutational tolerance or the breadth of the immune response would broadly differ between respiratory viruses and viruses undergoing systemic replication.

Here, we propose an alternative explanation for why some viruses undergo antigenic evolution while others do not. Our explanation is rooted in known differences between the immune response to viruses with replication limited to the respiratory tract versus to viruses undergoing systemic replication, and thus provides a more general explanation for observed differences between these two classes of viruses. In brief, immune responses to respiratory infections differ both qualitatively (antibody isotype and T-cell phenotype) and quantitatively (in their rate of waning) from those of systemic infections. This is because the response to respiratory viruses must cross from the blood into the respiratory tract. Resident memory T cells and antibodies of the immunoglobulin A (IgA) are actively transported from the blood to the respiratory tract where they control viral replication [7, 36]. Subsequent to clearance of the infection, high titers of antibodies and large populations of B and T cells are maintained systemically for many decades following infection or vaccination [2, 4]. In contrast to systemic immunity (which is long-lasting), immunity in the respiratory tract wanes rapidly with a half-life of weeks to months [20, 26, 27]. Consequently, reinfections with respiratory viruses can be observed even when the virus does not change antigenically [6, 14, 17], whereas reinfection with viruses that replicate systemically is rare [45]. Our model argues that this waning of immunity not only itself allows for reinfection, but that it is further a key driver of antigenic immune escape, which, in turn, exacerbates reinfection. Our model specifically proposes that partially immune hosts allow for immune escape variants to gain a selective advantage over a resident strain. These hosts, we propose, therefore play a key role in the *amplification* of escape variants at the level of the host population. As such, our model fundamentally differs from an earlier model [19] that proposed that partially immune hosts play a key role in the *generation* of escape variants at the within-host level.

## RESULTS

### Waning immunity drives viral evolution: intuition

At the host population level, waning immunity results in the generation of hosts with intermediate levels of immunity. The presence of these partially immune hosts can facilitate the emergence of viral immune escape in two distinct ways. The first is that immune escape variants may be produced at higher rates in partially immune individuals than in either solidly immune individuals or in naive hosts [19] (Fig. 1b). This argument posits that immune pressure in partially immune individuals is sufficiently low to allow viral replication while sufficiently high to impose selection pressure for immune escape. Viral replication is needed to enable the generation of *de novo* immune escape variants through the process of mutation [19, 28]. In this view, the population-level rate at which immune escape variants are generated is higher when the population comprises many partially immune individuals. This explanation, therefore, assumes that the emergence of antigenic escape variants is mutation-limited at the level of the host population.

A second, and yet unexplored, possibility is that waning immunity creates a population of partially immune hosts that enables the spread of immune escape variants at the level of the host population (Fig. 1c). These hosts serve as amplifiers of immune escape variants (regardless of which hosts they evolved in) because their presence results in a selective advantage of the immune escape variant over the circulating strain. The crux of this argument is shown in Fig. 1d, which depicts the within-host growth rate of two virus strains: a circulating strain that the host has previously been infected with (hereafter, the “wild-type” virus) and a newly emerging immune escape variant (hereafter, the “mutant” virus). Within-host growth rates are shown for individuals under three different levels of immunity (high, partial, and low), which individuals pass through following infection with the wild-type virus. Individuals having been infected recently have high levels of immunity. Within-host viral growth rates therefore fall below zero for both the wild-type and the mutant virus. As such, these individuals are not susceptible to infection with either the wild-type virus or the mutant virus. In contrast, individuals having been infected a long time ago have only low levels of immunity and within-host viral growth rates are therefore positive for both the wild-type and the mutant virus. These individuals are susceptible to infection with both the wild-type and the mutant virus and would be able to transmit these infections to others. We assume, however, that the mutant virus carries a replicative fitness cost and thus has a lower within-host viral growth rate than the wild-type virus in individuals with little to no immunity. This assumption is supported by a recent empirical study of H1N1 [41] and, in our model, manifests as a transmission advantage of the wild-type virus over the mutant virus in populations of hosts with little to no immunity.

The most critical component of our model centers on individuals with partial immunity. Individuals who were infected an intermediate length of time ago are partially immune, resulting in the within-host viral growth being negative for the wild-type virus but being positive for the mutant virus due to the mismatch between host immunity and the mutant virus’s antigenic phenotype. Partially immune hosts are thus not susceptible to infection with the wild-type virus but are susceptible to infection with the mutant virus. Waning immunity in this case has the potential to generate a class of partially immune individuals that can provide a selective advantage to the mutant virus at the level of the population, even if the mutation exacts a fitness cost. This explanation therefore assumes that the emergence of antigenic escape variants is not mutation limited at the level of the host population, but rather that it is selection limited.

While the mutation-limited and the selection-limited models are not mutually exclusive, they are quite distinct: the first argues that partially immune individuals increase the probability of generation of a mutant strain at the within-host level [19] whereas the second argues that partially immune individuals facilitate population-level transmission of the mutant virus. We consider here that this second possibility may be the primary driver of antigenic evolution for respiratory viruses. As such, we propose that waning of immunity in respiratory viruses and immune escape are not two independent factors that enable host reinfection, but rather that waning immunity also drives immune escape at the epidemiological level (Fig. 1c), placing waning immunity as the ultimately responsible culprit for respiratory virus reinfection.

In the present context, an abundance of hosts with partial immunity provides a subpopulation that enables growth of mutant strains that partially escape immunity. Those mutants can be thought of as “small-step” mutants because they could not spread in a population of just susceptible and immune hosts. In this model, the introduction of small-step viral mutants is not limiting, but their ascent in the population requires a sufficient density of hosts with partial immunity.

Attaining a “sufficient” abundance of partially immune hosts for a mutant virus to have a selective advantage rests on a combination of viral and host factors. Intuition suggests that, for the mutant virus to be able to invade, waning immunity rates should be neither too low nor too high. If immunity wanes too slowly, most recovered hosts will remain in the fully immune class, creating a shortage of partially immune hosts. If immunity wanes too quickly, recovered hosts will rapidly return to naïve status, where the wild-type virus has the transmission advantage. The basic reproduction number $R_{0}$ of the virus (a measure of transmission potential) is also likely to be important, as a low $R_{0}$ will lead to few infections and thus few recovered hosts, which are the precursors to partially immune hosts.

### Waning immunity drives viral evolution: a mathematical model

**Fig 2:**
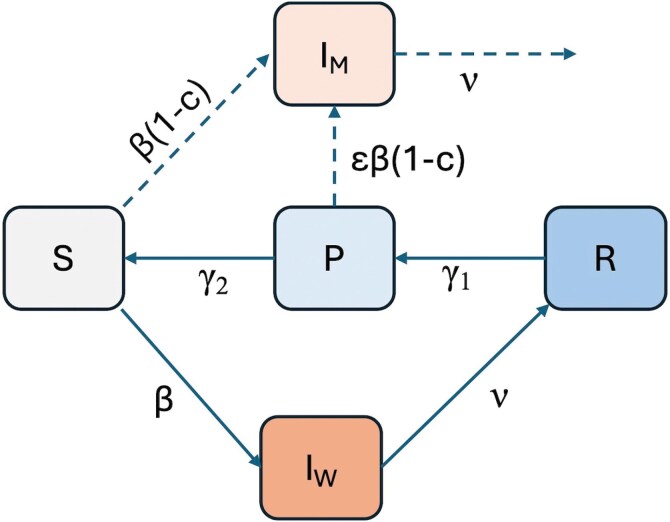
A schematic of the epidemiological model. Hosts are classified as susceptible to infection with the wild-type virus (S), currently infected with the wild-type virus ($I_{W}$), fully immune (R), or partially immune (P). Individuals infected with the mutant virus are shown in compartment $I_{M}$. The solid lines describe transitions involving infections with the wild-type virus. Immunity following infection wanes, with hosts first being fully immune (R) and not susceptible to infection with either the wild-type or the mutant virus. Hosts then transition to having partial immunity (P), which still protects from wild-type infection but not from infection with the mutant virus. Partially immune hosts then transition to being susceptible (S) to both the wild-type virus and the mutant virus. Dotted lines show transitions just after introduction of the mutant virus. The parameter c denotes the transmission fitness cost incurred by the mutant virus. The parameter ε denotes the extent to which partially immune hosts infected with the mutant virus are less infectious than susceptible hosts infected with this virus. For clarity, the schematic does not show births into compartment S or background mortality from any of the compartments.

To better understand this intuition, we introduce a simple epidemiological model with waning immunity (Fig. 2). We first consider the circulation of a wild-type virus and account for individuals who are susceptible to infection (S), individuals who are infected with the wild-type virus ($I_{W}$), individuals who have recently recovered and are fully immune (R), and (following [5]) individuals who are partially immune (P). Following recovery from infection, we let immunity wane gradually, with individuals passing in to and out of the partially immune host compartment at rates $\gamma_{1}$ and $\gamma_{2}$, respectively. We assume that only individuals in the S class are susceptible to infection with the wild-type virus. These individuals may never have been previously infected or they may have had a previous infection a long time ago (such that they have transitioned from R through P and back into S). We assume that individuals in the P class are not susceptible to infection with the wild-type virus nor are they boosted by exposure to individuals infected with the wild-type virus, distinguishing our model from that of [5].

The model is given by


$$\begin{array}{cccc} \frac{dS}{dt} & =\delta -\beta SI_{W}+\gamma_{2}P-\delta S & & \\ \frac{dI_{W}}{dt} & =\beta SI_{W}-\nu I_{W}-\delta I_{W} & \\ \frac{dR}{dt} & =\nu I_{W}-\gamma_{1}R-\delta R & & \\ \frac{dP}{dt} & =\gamma_{1}R-\gamma_{2}P-\delta P & & \end{array}$$ (1)

where variables S, $I_{W}$, R, and P represent proportions of the host population. Individuals enter the susceptible class through birth (δ) and through the loss of partial immunity ($\gamma_{2}P$) and leave this class through infection ($\beta SI_{W}$) and through background mortality (δS). Individuals enter the wild-type, infected class through infection ($\beta SI_{W}$) and leave this class through recovery ($\nu I_{W}$) and background mortality ($\delta I_{W}$). Individuals enter the fully immune class through recovery ($\nu I_{W}$) and leave this class as their full immunity status wanes ($\gamma_{1}R$) and background mortality (δR). Finally, individuals enter the partially immune class through waning of full immunity ($\gamma_{1}R$) and leave this class as their partial immunity status wanes ($\gamma_{2}P$) and background mortality (δP). Given this model structure, the basic reproduction number ($R_{0}$) for the wild-type strain is given by $R_{0}=\beta ∕(\delta +\nu )$ and the average duration of immunity to this strain is given by $T_{W}=1∕\gamma_{1}$ + $1∕\gamma_{2}$. We note that this model converges to the standard SIR model for the wild-type virus as this duration of immunity $T_{W}$ approaches infinity. This model also converges to the standard SIRS model for the wild-type virus either (a) as the average time spent in the R class ($1∕\gamma_{1}$) approaches 0 or (b) as the average time spent in the P class ($1∕\gamma_{2}$) approaches 0. Under scenario (a), the average duration of immunity to the wild-type virus is given by $1∕\gamma_{2}$. Under scenario (b), the average duration of immunity to the wild-type virus is given by $1∕\gamma_{1}$.

If $R_{0}<1$, there is a trivial equilibrium with only susceptible individuals present in the population. If $R_{0}>1$, there is an equilibrium with non-zero values for all classes $\left\{Ŝ,\hat{I_{W}},\hat{R},\hat{P}\right\}$. The equilibrium proportion of susceptible hosts is $Ŝ=(\delta +\nu )∕\beta =1∕R_{0}$. The equilibrium proportion of fully immune hosts is


$$\begin{array}{lll} \hat{R}=\frac{\nu }{\left(\gamma_{1}+\delta \right)}{Î}_{W}. & & \end{array}$$

The equilibrium proportion of partially immune hosts is


$$\begin{array}{lll} \hat{P}=\frac{\gamma_{1}}{\left(\gamma_{2}+\delta \right)}\hat{R}. & & \end{array}$$

Finally, the equilibrium proportion of wild-type virus infected hosts is


$$\begin{array}{lll} {Î}_{W}=\frac{\left(\gamma_{1}+\delta \right)\left(\gamma_{2}+\delta \right)(\beta -\delta -\nu )}{\beta \left(\gamma_{1}\nu +\gamma_{2}\nu +\gamma_{1}\gamma_{2}+\delta^{2}+\gamma_{1}\delta +\gamma_{2}\delta +\delta \nu \right)}. & & \end{array}$$

To consider the evolution of immune escape, we introduce infections with a mutant virus ($I_{M}$) into a population with the wild-type infection at equilibrium. This mutant virus can infect both susceptible hosts (S) and also partially immune hosts (P) because it differs antigenically from the wild-type virus. We assume that the escape mutation not only allows partial escape from immunity but also exacts a fitness cost c, consistent with its having a reduced within-host growth rate (Fig. 1d). Because a reduced transmission rate is a plausible consequence of the mutant virus’s lower within-host viral growth rate, we assume that the fitness cost c (0<c<1) of the antigenic mutation manifests as a lower transmission rate β, such that the mutant virus has a transmission rate of β(1−c). (The cost to the mutant could equally manifest as a faster recovery rate, such that the recovery rate of the mutant strain would be higher than ν.) Also as depicted in Fig. 1d, the mutant virus growth rate in partially immune hosts will likely be lower than in hosts with little to no immunity. We therefore model the possibility of a reduced transmission rate specifically from partially immune hosts using the parameter ε (0<ε≤1), which can reduce the transmission rate of partially immune hosts P carrying the mutant strain relative to that of susceptible hosts S carrying the mutant strain. With these assumptions in place, the dynamics of the mutant virus shortly following its introduction are given by


$$\begin{array}{ccc} \frac{dI_{M}}{dt}=\beta (1-c)(Ŝ+\varepsilon \hat{P})I_{M}-\nu I_{M}-\delta I_{M}. & & \end{array}$$ (2)

The mutant virus can invade at the level of the host population if its growth rate $r_{M}$ is positive, that is, when $\beta (1-c)(Ŝ+\varepsilon \hat{P})-(\nu +\delta )>0$. Equivalently, the mutant virus can invade if its effective reproduction number ($R_{M}$) exceeds 1. $R_{M}$ is given by


$$\begin{array}{ccc} R_{M}= & \frac{\beta (1-c)(Ŝ+\varepsilon \hat{P})}{\nu +\delta }. & \end{array}$$ (3)

With the equilibrium number of susceptible individuals in the population being $Ŝ=1∕R_{0}$, this expression can be simplified to


$$\begin{array}{ccc} R_{M}= & (1-c)(1+R_{0}\varepsilon \hat{P}). & \end{array}$$ (4)

From this equation, we can see that the invasion potential of the mutant strain is entirely determined by its fitness cost c, the basic reproduction number $R_{0}$ of the wild-type virus, the extent to which transmission of the mutant strain is lower in partially immune hosts compared with hosts with little to no immunity ε, and the equilibrium density of partially immune hosts $\hat{P}$. The equilibrium density $\hat{P}$ is affected by multiple parameters, especially the immune decay parameters $\gamma_{1}$ and $\gamma_{2}$. These parameters are specific to the mutant virus because they quantify when a recovered host becomes susceptible and how long it remains immune to homologous reinfection. These parameters also reflect the magnitude of antigenic changes in the mutant. For example, mutant viruses that are antigenically more distinct from a wild-type virus should be able to reinfect recently recovered individuals more quickly, reflected in a larger $\gamma_{1}$ value.

**Fig 3:**
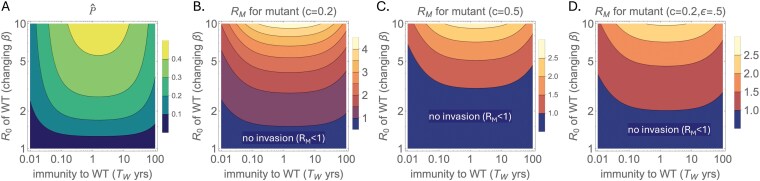
Invasion potential of a mutant virus that can infect partially immune hosts. (a) Equilibrium density of partially immune hosts $\hat{P}$ when the wild-type virus is at endemic equilibrium. $\hat{P}$ is shown as a function of two parameters: the $R_{0}$ of the wild-type virus and the average duration of immunity to reinfection with the wild-type virus $T_{W}$. Changes in $R_{0}$ are implemented as changes in the transmission rate β. The average duration of immunity to reinfection with the wild-type virus is given by $T_{W}=(1∕\gamma_{1}+1∕\gamma_{2})$. Here, across all $T_{W}$, we let the average duration of immunity to the mutant ($T_{M}$) be half of that to the wild-type virus: $1∕\gamma_{1}=(1∕2)(1∕\gamma_{1}+1∕\gamma_{2})$, which results in $\gamma_{1}=\gamma_{2}$. (b–d) Calculated values of the mutant virus’s effective reproduction number $R_{M}$ as a function of the wild-type virus’s $R_{0}$ and $T_{W}$. In (b), the fitness cost is c=0.2 and ε=1. In (c), the fitness cost is c=0.5 and ε=1. In (d), the fitness cost is c=0.2 (as in (b)) and ε=0.5. Note that log scales are used on both axes in panels (a–d). In all panels, δ= 1/50 years^−1^ and ν= 1/7 days^−1^.

In Fig. 3a, we first plot $\hat{P}$ as a function of two parameters of the epidemiological model shown in Fig. 2: the average duration of immunity to wild-type virus reinfection ($T_{W}=1∕\gamma_{1}+1∕\gamma_{2}$, where we assume $\gamma_{1}=\gamma_{2}$) and the wild-type virus’s $R_{0}$. The plot shows that the proportion of partially immune hosts is highest at high $R_{0}$ and at an intermediate duration of immunity. As per equation (4), this should also be the region most favorable to invasion by the mutant strain (that is, where $R_{M}$ is highest). Indeed, when we plot $R_{M}$ as a function of these same two parameters under two different fitness costs (c=0.2 in Fig. 3b and c=0.5 in Fig. 3c, in both cases assuming ε=1), we see a broadly similar relationship between $R_{M}$ and these underlying parameters. In Fig. 3d, we relax the assumption of ε=1 and plot $R_{M}$ when the fitness cost is again c=0.2 and partially immune hosts infected with the mutant virus are only 50% as infectious as susceptible hosts infected with the mutant virus (ε=0.50). In all three panels, we find that there is a zone defined by values of $R_{0}$ and the duration of immunity above which the mutant virus can invade.

Owing to the direct effect of $R_{0}$ on the invasion potential of the mutant, $R_{M}$ increases with the $R_{0}$ of the viral infection, in agreement with the intuition described in the previous section. As is evident from equation (4), a higher fitness cost c results in a direct reduction in $R_{M}$, such that the region of parameter space where the mutant can invade is smaller at higher costs (Fig. 3c versus Fig. 3b). A comparison between Fig. 3d and Fig. 3b demonstrates that a smaller value of ε also results in a reduction in $R_{M}$, particularly at high $\hat{P}$ values, such that the region of parameter space where the mutant can invade is smaller when partially immune hosts transmit the mutant strain less well than susceptible hosts transmit the mutant strain. This outcome is also evident from equation (4).

**Fig 4:**
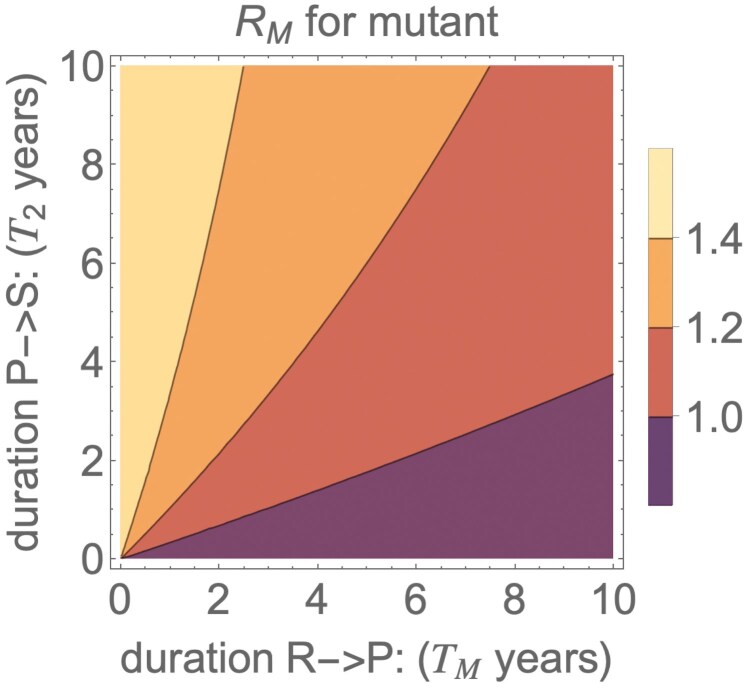
Invasion potential depends on the time $T_{M}$ taken for individuals to go from *R* to *P* (fully to partially immune) vs the time $T_{2}$ to go from P to S (partially immune to fully susceptible). $T_{M}=1∕\gamma_{1}$ is the average duration of time an individual stays in the R host class and thus remains immune to infection with both wild type and mutant virus. $T_{2}=1∕\gamma_{2}$ is the average duration of time an individual remains in the P host class, where they are immune to infection with the wild-type virus but susceptible to infection with the mutant virus. Here, $R_{0}=2$, c=0.2, and ε=1.

Fig. 3 showed how $R_{0}$ and $T_{W}$ affect the invasion potential of a mutant virus, assuming that the rate of transitioning from R to P was the same as the rate of transitioning from P to S ($\gamma_{1}=\gamma_{2}$.) The invasion potential of the mutant virus, however, does not only depend on $T_{W}$ but depends on the average duration of immunity following a wild-type infection that a mutant virus sees, which we denote as $T_{M}$. Given the model structure, $T_{M}$ is given by $1∕\gamma_{1}$. Fig. 4 shows how the invasion potential of the mutant virus depends on the durations of immunity to the mutant virus ($T_{M}$) and the time an individual remains partially immune ($T_{2}=1∕\gamma_{2}$, the average time an individual is susceptible to infection with the mutant virus but not the wild-type virus). The invasion potential of the mutant virus $R_{M}$ is highest when there is a fast waning of “early” immunity (small $T_{M}$) and a slow waning of “late” immunity (large $T_{2}$). This asymmetry makes sense because a small $T_{M}$ quickly converts recovered hosts into hosts that can be infected only by the mutant virus, and a large $T_{2}$ slows the rate at which those hosts again become susceptible to infection with the wild-type virus.

The model presented here merely considers the effect of waning immunity on the invasion of initial immune escape variants. We envisage that at the outset (when the wild-type virus first invades), immune escape variants will initially have a net fitness disadvantage relative to the wild type, because the antigenic escape has a fitness cost, and there is yet little to no immunity built up to the wild-type virus in the host population. However, as the wild-type virus becomes endemic, full and partially immune hosts will accumulate toward their equilibrium values. As this happens, $R_{M}$ will start to exceed 1, and the mutant virus will invade. After the mutant virus invades, we expect that subsequent compensatory mutations will be rapidly generated and reduce the fitness cost. As a result, the basic reproduction number of the mutant virus will approach that of the wild-type virus, such that no progressive decline in circulating viral fitness is expected during the successive emergence of new antigenic variants and the replacement of older viral lineages.

## DISCUSSION

It is now well appreciated that immunity to many respiratory viruses wanes rapidly. We have suggested here that this waning immunity facilitates the evolution of viral immune escape. Our model posits that an abundance of hosts with partial immunity provides an environment that allows immune escape variants a population-wide transmission advantage over a wild-type strain when these escape variants would be unable to spread in the absence of these partially immune hosts. Thus respiratory viruses can undergo rapid antigenic evolution, whereas viruses that undergo systemic replication do not. Our model is motivated by the observation that immunity wanes for respiratory viruses, presumably due to their site of replication, whereas immunity is longer lived for systemically replicating viruses.

Indeed, none of the most common respiratory viruses affecting humans (influenza viruses, coronaviruses, metapneumonia virus, parainfluenza viruses, RSV, and rhinovirus) result in durable immunity [45], and all exhibit antigenic evolution. In comparison, many viruses that undergo airborne transmission but replicate systemically do result in durable immunity [45], including ebola, measles, mumps, parvovirus, rubella, varicella, and variola viruses. None of these latter viruses exhibit antigenic evolution.

Along a similar line of argument, some fecal-orally transmitted viruses that infect the gastrointestinal (GI) tract also do not undergo systemic replication and do not induce durable immunity (noroviruses and rotaviruses) [45]. In line with our expectation, those viruses evolve antigenically. These broad patterns support the generality of our model, which argues that waning immunity (immunity that is not durable) drives the spread of immune escape variants.

Currently, we are not aware of any examples that would invalidate our model and only one exception that violates the pattern of durable immunity and respiratory or GI replication. The possible exception is poliovirus, a GI pathogen that is antigenically stable and which elicits lifelong immunity [45]. Polio disease results from viral replication outside the GI tract, but viral transmission likely requires only GI replication [8, 29, 37]. The pattern of antigenic stability and absence of waning immunity fits our model, but the absence of waning immunity does not fit the pattern that viruses infecting and replicating in mucosal tissues exhibit waning immunity. Specific to our model, future work could focus on identifying respiratory or GI tract viral pathogens where immunity wanes but where antigenic evolution is inapparent, or identifying viral pathogens that undergo systemic replication and exhibit antigenic evolution at the level of the host population. Examination of such exceptions would be instructive for testing and refining our proposed model of waning immunity as a driver of immune escape. Understanding why mucosal immunity is waning for some viruses but not others is also an important area to explore further.

The model presented here has been deliberately simplified to show the effect of waning immunity on the ability of immune escape mutants to invade. The primary reason for this simplicity is the lack of quantitative knowledge of the parameters governing the waning of immunity (particularly in the respiratory tract) as well as the fitness costs of immune escape mutations. Under these circumstances the simple model allows us to get an intuitive understanding of the problem under investigation, namely the role of waning immunity for the emergence of antigenic variants. The model as it stands serves to identify key features and parameters that warrant detailed investigation in future studies.

Our study focused on antigenic evolution that is observed over time in respiratory viruses causing acute infections at the host population level. Because it is a simple model that considers only a single mutant virus and its ability to invade a population with an endemic wild-type virus, it cannot be used to predict the timescale of antigenic evolution, or whether antigenic diversification will occur in the long-run, versus only successive emergence and replacement of antigenic clusters. Other model structures, such as a recent one developed by Park *et al.* [34], are needed to address this question. Finally, because it is not a mutation-limited model, our model also does not speak to the sources of new antigenic variants, whether those might be partially immune individuals or individuals who are persistently infected. (Of note, our model also does not pertain to understanding antigenic evolution in persistent infections, whether of systemically replicating viral pathogens such as HIV and HCV or respiratory viruses such as influenza viruses or coronaviruses in the case of immunosuppression/immunocompromise.)

We now turn to the relationship of our study to prior work. Grenfell *et al.* [19] suggested that partly immune hosts are required for the within-host evolution of mutants that escape immunity. If these escape variants do not have a fitness cost, they will then have a selective advantage in the host population by being able to infect hosts with full immunity to the wild-type virus. A distinction between our model and theirs is that, in ours, the generation of mutants within a host is not limiting and the mutations initially are associated with a fitness cost. In our model, escape mutations are assumed to be generated frequently (albeit with some initial fitness cost), and it is the widespread prevalence of partially immune hosts that allows for (and is required for) these escape-variants to invade. Thus, under our model, the initial variant that spreads may, nonetheless, be unable to infect hosts with full immunity. It is worth noting that within-host evolution of escape variants and the mechanism proposed here are not mutually exclusive: both can operate. Another previous study proposed a model to explain the pattern of strain replacement that gives rise to the ladder-like phylogeny of viruses such as that of influenza A subtype H3N2’s hemagglutinin gene [13]. In that study, the authors invoked a long-lived strain-specific immunity and a short-lived strain-transcending immunity. Waning immunity provides a specific mechanism to achieve both long-lived (strain-specific) immunity and short-lived (strain-transcending) immunity and does so on a continuous scale. Our model invokes both “types” of immunity: recovered hosts have strain-transcending immunity but they decay into partially immune hosts with strain-specific immunity.

Alternative mechanisms for antigenic evolution—and for antigenic stability—have been proposed. We describe these by contrasting respiratory viruses such as influenza with the measles virus, which is so antigenically stable that the same vaccine has been effective for over 50 years. As mentioned by Yewdell [45], although measles is transmitted by the respiratory route, its replication occurs systemically, predominantly in B cells. Systemic immunity blocks systemic replication and prevents transmission even if pulmonary immunity has waned. From the perspective of our model, measles does not experience waning immunity because once-infected hosts remain protected against infection for life.

One alternative explanation for antigenic stability of viruses such as measles is merely that the proteins targeted by immunity are constrained from changing—the cost of immune escape is too high to evolve [18, 42]. This hypothesis does not explain a systematic difference between respiratory viruses and those that cause systemic infections.

A more intriguing possibility has been suggested for measles [18]: measles virus fails to escape immunity because it elicits multiple codominant responses to several epitopes on the virus. That paper also posited that influenza escapes because the immune response focuses on a single immunodominant epitope. We agree that multiple codominant responses limit the opportunities for antigenic escape—by increasing the number of mutations required to overcome immunity. But this argument may work only if immunity remains high to multiple epitopes. Our view is that, regardless of whether the response to influenza targets single or multiple epitopes ([3] provides evidence of multiple epitopes), escape evolves because waning immunity to all epitopes eventually exposes the virus to a selective regime where escape mutants will be selected.

Waning immunity may have consequences for the long-term fate of universal vaccines [12, 31]. Universal vaccines need to target highly conserved protein epitopes, so that immunity protects broadly. However, with waning immunity, an escape variant may inevitably arise and spread unless those epitopes are subject to sufficiently strong steric constraint as to impose a prohibitively high fitness cost to the mutant. Regardless, waning immunity will require the vaccine to be given repeatedly. Thus, at best, a universal vaccine developed for a virus with waning immunity is one that does not need to be reformulated but it will require frequent booster shots.

Our model can be refined in a number of ways such as by considering the gradual waning of immunity (rather than a single class of partial immunity) and how this waning affects recall responses. Doing so will require a quantitative understanding of measures of immune efficacy that include susceptibility to infection as well as the level of transmission from infected individuals. We caution that waning immunity does not ensure the evolution of escape, rather the outcome depends on quantitative issues of how much and how fast immunity wanes as well as the fitness costs experienced by escape mutants. Waning immunity merely facilitates the evolution of escape.

Our study has addressed a small problem in the grand picture of viral evolution and phylodynamics. Even if waning immunity proves to be a driver of viral antigenic evolution on a small, short-term scale, there remain many puzzles in which this result should be integrated. Some of these have been alluded to in preceding sections and include evolution beyond invasion and integrating the evolutionary and epidemiological scales to understand different patterns of antigenic evolution and phylodynamics of different respiratory infections. It will thus be important to understand whether our model is compatible with and can account for observed patterns of viral evolution over the long term.

## Data Availability

All data used to create Figs. Fig. 3 and Fig. 4 were generated with simulations of the appropriate equations in the paper.
